# The contribution of blog-based writing instruction to enhancing writing performance and writing motivation of Chinese EFL learners

**DOI:** 10.3389/fpsyg.2022.1069585

**Published:** 2023-01-18

**Authors:** Shujun Han

**Affiliations:** Henan University of Animal Husbandry and Economy, Zhengzhou, Henan, China

**Keywords:** technology, writing skill, blog-based writing, writing motivation, writing assignments, EFL instructors

## Abstract

As an attempt to shed more light on the utility of blogs in educational contexts, the current research set out to explore the effectiveness of blog-based writing instruction on the writing skill and writing motivation of Chinese English-as-a-Foreign Language (EFL) students. Forty-nine Chinese EFL students from two intact classes in a language school were selected as the participants through convenience sampling. Then they were assigned to an experimental (26 students) and a control condition (23 students) randomly. The students of the experimental group utilized blogs for posting their writing assignments whereas the control group were taught traditionally with no use of blogs or other technology devices. The IELTS writing tasks and L2 writing motivation scale were used for the data collection as pre-and post-tests. The findings revealed that although both groups improved their writing skill and motivation, the participants of the blog group performed better than those in the control group, confirming that the blog-based teaching substantially enhanced the writing skill and writing motivation of the EFL students. These findings may have notable implications for EFL instructors.

## Introduction

Nowadays, the arrival of novel technology applications has triggered a renewal of surge of attention to the use of technology in education contexts (Smith et al., 2020). The widespread internet growth has affected a considerable increase in technology use recently. Numerous studies have proven technology used to be an effective tool for improving students’ motivation, autonomy, and creative thinking (Bain and Ross, 2000; Annetta et al., 2009; Arslan and Şahin-Kızıl, 2010; Sabzian et al., 2013; Yurtseven et al., 2015; Liu et al., 2022; Vu et al., 2022). In light of the positive impact that technology has had on students, the integration of technology in education has been the primary concern of many instructors (Hew and Brush, 2007; Hung et al., 2022; Teo et al., 2022; Yang et al., 2022). Communication is one of the key aspects of using technology, as it provides worldwide connections among friends, classmates, and experts. As a result of the growth in communication, students are exposed to greater learning opportunities (Sun, 2010).

Recently, the use of technology has undergone pioneer expansion in L2 learning (Du and Wagner, 2007; Kukulska-Hulme et al., 2017; Fathi and Rahimi, 2022). With the aid of technology, learners become active content creators (Du and Wagner, 2007). Technology also helps learners have access to an unlimited range of authentic materials with no restrictions (Hubbard et al., 2004). It fosters genuine communication among peers and teachers and increases the sense of responsibility and ownership (Murray and Hourigan, 2008; Dippold, 2009; Sun, 2009; Arslan and Şahin-Kızıl, 2010). Owing to the arrival of Web 2.0 technologies and widely uses of various applications, students’ exposure to L2 materials has increased (Wang and Vásquez, 2012; Lee and Markey, 2014). Therefore, students’ motivation and learning attitudes are enhanced (Fathi et al., 2019), and students with busy schedules make better use of their time by being able to work at home (Raja and Nagasubramani, 2018). Web 2.0 technology provides language learners with an effective and cooperative context in which they access unlimited information and feel a sense of responsibility for their own learning (Campbell, 2003; Lee, 2008). Eventually, the prevalent use of the internet in this age of technology has made educators more aware of the crucial role of blogs in education (Liu, 2016; Su et al., 2018).

As far as educational technology devices are concerned, a notable example of web 2.0 technology is blogs. Blogs are one of the most widely used websites for language learning. These blogs are easy to use with a convenient and easily accessed design (Bartlett-Bragg, 2003; Lee, 2008; Elola and Oskoz, 2017). Blogging websites provide learners with unlimited opportunities to get involved in authentic communication (Ducate and Lomicka, 2005; Richardson, 2006; Arena, 2008; Murray and Hourigan, 2008; Lee, 2020). In addition, with the help of blog-based writing, teacher feedback and peer feedback are enhanced (Campbell, 2003; Murray and Hourigan, 2008; Lee, 2010). Concerning L2 learning, a number of researchers have underscored the usefulness of blogging in enhancing writing competencies (e.g., Sun, 2010; Chen, 2016; Lee, 2017; Yousefifard and Fathi, 2021). In L2 writing context, blogs can provide convenient access to various online resources, foster greater writing practice, offer a real audience for the essays, enhance cooperative learning, generate a sense of community, ownership, as well as autonomy among L2 learners, and increase teacher–student and peer interactions in and outside the classroom (Armstrong and Retterer, 2008; Dippold, 2009; Chen, 2016; Özdemir and Aydin, 2020). It is also worth noting that WordPress, Blogspot, and Blogger are well-known website creation platforms used for L2 learning. By the use of blogs, learners can upload files and comment on the uploaded files. Commenting feature improves learners’ writing skills according to teacher feedback and peer feedback, and it additionally enhances self-confidence and self-efficacy. As a result of increasing interest in blogging, learners become aware of their strengths and weaknesses through receiving feedback, revising their writings, and finally, learners become creative writers (Murray and Hourigan, 2008; Lee, 2010). Although a significant body of studies has investigated the use of blogs in education in general and in L2 writing in particular (Armstrong and Retterer, 2008; Pham and Usaha, 2016; Lee, 2017; Yousefifard and Fathi, 2021), this research area calls for further empirical studies. Additionally, due to the complexity of writing nature as well as various topics, teaching methods, and wide scope, carrying out replication studies are much needed in L2 writing research (Porte and Richards, 2012). Furthermore, concerning writing skill, motivation is deemed an effective psychological construct that affects L2 writing significantly (Kormos, 2012; Wang and Guan, 2020). However, L2 writing motivation seems to be less researched in the body of related literature (Waller and Papi, 2017; Yousefifard and Fathi, 2021). As a result, to bridge the gap, this research is set to investigate the contribution of blog-based writing instruction to enhancing the writing performance and writing motivation of EFL learners. Therefore, the following two research questions were addressed in this study:

RQ1: To what extent does blog-based writing instruction affect writing performance of Chinese EFL students?

RQ2: To what extent does blog-based writing instruction affect writing motivation of Chinese EFL students?

## Review of literature

The theoretical framework guiding the effectiveness of Web 2.0 technology in L2 learning in the present study is Vygotsky’s (1978) socio-constructivist theory of learning. According to this theory, learners are able to perform learning tasks more effectively when they collaboratively or jointly construct knowledge (Pavlenko and Lantolf, 2000). According to this theory, when students complete a task, they act as peers and either implicitly (indirectly) or explicitly (directly) intervene and scaffold each other’s learning performance. This mechanism can aid peers in developing through their zone of proximal development (ZPD) where learning and advancement are connected (Lee, 2008). From Vygotsky’s (1978) stance, ZPD is concerned with the distance between individuals’ actual level of autonomous performance without receiving help or guidance and their potential development achieved under guided direction or as a team member with additional proficient peers.

Mediated learning or other-regulation is also essential and interconnected notions in Vygotsky’s (1978) theory of learning. Based on Lantolf and Thorne’s (2006) views, mediated learning is concerned with a cycle wherein learners or teachers control other students’ or their own social and mental abilities through socially developed ideas and activities. Learners are believed to be at first mediated by other social and group individuals as well as actions which in Vygotsky’s conceptualisation is known as other-regulation. The skills which initially lie in learners’ social collaborations slowly become internalized through which learners can self-mediate or self-control their cognitive capacities (Vygotsky, 1986). Blog, as a kind of Web 2.0 technology, provides the participants of this study to have collaboration and interaction in exchanging ideas about their essays, thereby fostering mediated learning or other-regulations among EFL writers. More particularly, through such a social joint effort/collaboration, learners can co-build L2 information; for instance, L2 composing with different peers, a process which improves L2 writers’ cognitive functioning and assists them with arriving at their possible level of functioning (Neumann and McDonough, 2015; Hung and Nguyen, 2022).

### Blogs in second language learning

During the last few years, Web 2.0 technology has become increasingly prevalent in L2 learning and instruction. A large and growing body of literature has investigated the effectiveness of blogs in L2 writing pedagogy (Stevenson and Liu, 2010; Amir et al., 2011; Trajtemberg and Yiakoumetti, 2011; Wang and Vásquez, 2012; Vurdien, 2013; Hansen, 2016; Lee, 2020; Yousefifard and Fathi, 2021; Zhang and Zou, 2021). The use of blogging to enhance teaching and learning has grabbed the attention of many language experts (de Almeida Soares, 2008). The purpose of this section is to review a few more relevant studies that examine blogging approaches in L2 writing.

For example, Lee (2010) used blogs as out-of-class assignments to develop learners’ language abilities. Seventeen university students at advanced levels kept blogs for 14 weeks as part of the study. The data was collected from blog pages and in final interviews, participants reported the advantages and challenges of using blogs for personal expression and interactions on a collaborative basis. As a result of regularly creating blog entries, learners’ writing fluency and motivation to write for various audiences are enhanced. Additionally, peer feedback led to further discussion, and feedback from the trainer led to more focus on language accuracy. Finally, the results showed that students’ critical thinking is crucial for applying blogging to the learning process.

In another study, Vurdien (2013) carried out a study to explore how a blog as a computer-mediated tool engages a group of EFL learners at a language school in Spain in reflective and collaborative learning. This study lasted for 5 months and the participants were 11 students preparing for the Cambridge examination. Activities like letter writing, proposal, and article writing were given to students. According to the collected data from discussions, feedback, and questionnaires, students’ collaborative interaction and self-reflection were fostered. Ducate and Lomicka (2008) carried out a one-year study to examine the students’ overall perceptions of blogging and their improvement in both reading and writing blogs. The collected data from blogs, reports, and interviews indicated that blogging enhanced students’ ownership and ingenuity in a peaceful situation.

To elaborate more on the studies concerning blogging, Trajtemberg and Yiakoumetti (2011) investigated the interaction strategies bloggers use by exploring both teacher-initiated and student-initiated interactions. The participants of this study were from an EFL undergraduate class at the University of Chile. Posts and comments were analyzed and the findings suggested that students’ self-awareness and self-reflection were fostered, moreover, their language learning enhanced. To increase the existing knowledge of blogging in second language learning, Amir et al. (2011) carried out a mixed methods study to investigate the use of blogs as an aid to develop students’ collaborative writing. The participants of this study were 4 classes of 80 students majoring in English Language Studies (ELS) programs and Literature programs. The results of this investigation showed that blogging gives students numerous options to write, interact and promote their sense of self-governance. In addition, different learning skills are given to students to foster self-confidence and keep learning in an authentic and peaceful place.

Conducting a quasi-experimental research, Arslan and Şahin-Kızıl (2010) verified the effectiveness of blog-based writing, using a process writing approach, on the writing skills of EFL students. Likewise, Vurdien (2013) investigated the impact of blogging technique in the writing course for EFL students who were requested to use blogs for posting their assignments and give feedback to their peers. The findings revealed that blog-based writing teaching improved the writing motivation of EFL learners. By the same token, Lee (2010) confirmed the utility of employing blogs in enhancing the writing skills and motivated behavior of EFL learners. In another study, Fathi et al. (2019) carried out an explanatory sequential design to probe the effects of blogs on writing affective variables of EFL learners. The results indicated that blog-based writing teaching was effective in enhancing writing motivation and writing self-regulation of the EFL learners. However, they found that blog-based writing courses diminished the writing self-efficacy of the participants. Özdemir and Aydin (2020) also averred the effectiveness of blog-based writing instruction on L2 motivation in a Turkish EFL context. Regarding affective variables, Chen (2016) also found that blogging failed to have a substantial effect on the writing motivation and anxiety of EFL learners although it improved their writing self-efficacy significantly. Yousefifard and Fathi (2021) also found that blog-based writing instruction significantly improved writing abilities and the ideal writing self, as a key component of writing motivation, among EFL students.

Also, Fageeh (2011) investigated the implementation of blog in an intermediate EFL writing class and its influence on students’ insights and writing instruction. A triangulated research strategy was conducted to study the effects of blogging on writing proficiency and attitude through a combination of an experimental and descriptive research design. The participants of this study were college students majoring in language translation. Twenty-five students were involved in the experimental group and the other 25 students were assigned to the control group. According to the results, the students viewed Weblog as a way to improve their writing skills and attitudes toward writing. Generally, students showed positive perspectives toward weblogs and they also believed that weblogs give them numerous opportunities to improve autonomy, self-governance, and creative writing.

According to another study by Hansen (2016), the effectiveness of blog-style writing tasks in comparison with more traditional writing tasks was analyzed. A technical analysis was conducted to compare the two different writing styles. Also, a self-assessment analysis was conducted to determine whether the tasks influenced further interest in the topic and whether they were engaging with the class’s learning objectives. In addition to that, as a part of the evaluation process, the amount of spent time on each task, the number of prepared drafts, and the types of the sources being used were analyzed. Despite the data collection limitations in this study, preliminary evidence suggested that blogs and essays work best when combined as supplementary writing assignments, while each encourages students to develop different abilities.

To elaborate more on the studies concerning blogs, further research on this topic was conducted by Lee (2020), to investigate the effect of using blogs in enhancing L2 writing skills in online language courses. The participants of this study were 48 beginner students who used blogs for writing tasks for 2 years. The data involved both qualitative and quantitative data and it was gathered from blog posts, comments, and last interviews. The findings of this research showed that students perceived blogging as a powerful method for enhancing their writing abilities. Additionally, the results revealed that students’ efforts to boost both form and content were affected by feedback scaffolding. It is also concluded that in order to encourage reflective thinking and collaborative learning, sufficient time should be allocated for feedback interaction, and strategies for effective use of feedback.

### Writing motivation

The findings obtained from numerous studies have indicated that motivation counts as a significant part of language learning (Oxford and Shearin, 1994; Williams and Burden, 1997; Williams et al., 2002; Waller and Papi, 2017; Bui and Nguyen, 2022). Motivation in L2 writing is based on the general construct of motivation in L2 education (Waller and Papi, 2017; Fan and Wang, 2022). Dörnyei (2001) describes motivation as a dynamic process susceptible to continuous change as an integral part of successful L2 learning. Williams and Burden (1997) indicated that L2 motivation is influenced by external factors relevant to a learner’s sociocultural background and internal factors which are unique to each individual learner. Moreover, learning motivation is a combination of effort and a desire to learn plus favorable attitudes toward learning (Gardner et al., 1985; Fu and Wang, 2022). Writing motivation is important since writers express themselves, communicate with others, and appeal to people (Chen, 2016). Some researchers have explored the factors contributing to L2 writing motivation. For instance, Shen et al. (2020) found an intricate interconnection between task value and self-efficacy, as two key motivational constructs, with regard to EFL writing among Hong Kong primary students. They considered these two constructs as the significant predictors of writing motivation which in turn could enhance L2 writing outcomes. Additionally, some researchers have attributed L2 writing motivation to such factors as feedback type, proficiency level, students’ attitudes, and learning experience (Han and Hyland, 2015; Zheng and Yu, 2018; Yu et al., 2020). Likewise, Han and Hiver (2018) found that that L2 writers’ motivation could be improved using the genre-based writing teaching. Lee et al. (2018) reported that proficiency level and grade were among the factors that contributed to writing motivation of EFL students in Hong Kong. Also, having carried out a large-scale research, Yu et al. (2019) found that EFL motivation as well as engagement of Chinese undergraduate students was relatively high and individual differences were influential in affecting writing motivation of these participants.

Concerning the effect of blogging on L2 writing motivation, several studies investigating writing motivation have been carried out that will be reviewed here. For example, Fathi et al. (2019) investigated the effects of blogging on L2 students’ writing motivation. The participants of this study were 46 Iranian EFL students. A number of 21 students were assigned to the control group, and the other 21 students were in the experimental group. This study lasted for 16 weeks, and the control group received traditional writing instruction, on the other hand, the experimental group received blog-mediated writing instruction. By the use of writing instructions and interviews data were collected; afterward, ANCOVA and data coding was administered for data analyses. The results of this research showed that blogging helped EFL students have more writing motivation; however, blogging reduced students’ writing self-efficacy. In another study, Montero-Fleta and Pérez-Sabater (2010) carried out research to investigate the effect of blogging on language skills. The participants were 38 Spanish students with an average age of 23.6 and intermediate level. The control group contained 19 students receiving traditional instruction and the experiment group contained 19 students receiving blog-based writing practices. The results approved the positive effects of blogging as mentioned by Arena (2008). Moreover, the results firmly confirmed the increase in motivation while blogging. As a result of blogging, students become motivated to creatively write for a large amount of authentic audience.

Additionally, Chen (2016) conducted a quasi-experimental study to analyze the EFL learners’ metalinguistic and affective performance as a result of blogging. The results indicated that in terms of metalinguistic strategy use, there were no significant differences between the two groups, but in terms of metalinguistic awareness, there were significant differences. Among the affective variables, writing motivation and writing anxiety were identical between groups. In terms of self-efficacy, however, the control group significantly outperformed the experimental group. Furthermore, Özdemir and Aydin (2020) investigated the effect of blogging on Turkish EFL learners’ writing motivation. The participants of this study were 48 language learners who were given a questionnaire that inquired about their demographics and a pre-test and post-test that assessed their writing ability. According to their findings, blogs alone were not sufficient to enhance students’ writing motivation; however, a process-based writing approach significantly enhanced motivation.

## Methodology

### Participants

To accomplish the goals of this research, a quasi-experimental design was used. Forty-nine intermediate Chinese EFL students were selected using convenience sampling. This method of sampling was used as the researchers were not able to use probability sampling methods and they had more access to these available participants who were also willing to participate in the research. These participants were, in fact, students from two intact classes in the central part of the Chinese Mainland. The classes included mixed-gender students and were assigned to an experimental (26 students) and a control condition (23 students) randomly. The age of these EFL learners varied from 19 to 25 and they had the learning experience of English ranging from 6 to 12 years. The participants had enrolled in a preparation English program whose aim was to foster the writing skills required on IELTS test tasks. Both groups were instructed by the same practitioner who was an experienced IELTS teacher and familiar with educational blogging. Nevertheless, the participants mentioned that they had never used blogs for learning purposes before.

### Instruments

#### English homogeneity test

The English proficiency of the participants was assessed *via* an online DIALANG test. DIALANG is a test of general language proficiency which evaluates the testees language proficiency from A1 to C2, in line with the Common European Framework of Reference for Languages (CEFR). This online test measures the three skills of reading, writing, and listening. It also measures grammar and vocabulary components. The mean scores of the two groups on the DIALANG test were compared by running an independent samples t-test whose results indicated that there was no significant difference between the groups, revealing their homogeneity in general English proficiency.

#### Writing performance test

IELTS samples of academic writing task 1 were used to measure EFL students’ academic writing competencies, as pre-test and post-test. The writing tasks were selected from ‘Collins writing for IELTS’ (Williams, 2011). In addition, the academic writing competencies of the participants were rated against the IELTS academic writing scale and descriptors (University of Cambridge ESOL Examinations, 2011). To ensure the inter-rater reliability of the scoring, an independent rater who was familiar with IELTS writing tasks marked the written tasks of the students. The agreement coefficient between the two raters’ scores on academic writing tasks was high (*r* = 0.88).

#### Writing motivation scale

Participants’ L2 writing motivation was measured using the questionnaire developed by Waller and Papi (2017). This self-report scale evaluates EFL learners’ motivation and efforts in writing, writing desire, motivational intensity for L2 writing, teaching feedback, organization and content, and peer feedback. This questionnaire includes seven items which are assessed on a 5-point Likert scale from 1 (never) to 5 (always). The internal consistency of the scale in this research, as estimated by Cronbach’s Alpha formula, turned out to be high (*r* = 0.88).

### Procedure

The first session of the course was devoted to administering the pre-tests and the English proficiency test (i.e., DIALANG). After making sure about the homogeneity of the participants in terms of English proficiency test, the two intact classes were randomly assigned to an experimental and a control group.

The purpose of this writing course was to enhance students’ academic writing as discussed earlier. Within a course of 12 weeks, the participants were provided with practical strategies and content to boost their writing skills. In sum, they were instructed on how to develop ideas, write different types of paragraphs, outline a text, and revise it.

As regards the experimental group, the teacher created a class blog for the students and held a trial session for them to familiarize them on how to use the blog and post their essays on it. There were hyperlinks of the useful ESL websites on the class blog and the students could use these websites to get exposed to various types of paragraphs and the useful supplementary vocabulary and grammar materials they could use in doing their written tasks. The students of the experimental group were required to publish their written tasks on the blog regularly, share ideas on the writing tasks, and give feedback to their classmates. More precisely, each student was requested to post at least two writing tasks on the class blog and also publish at least five comments per week. The tasks ranged from 350 to 800 words and were assigned by the instructors based on the coursebook as well as the covered content of each session. The students’ comments were also concerned with content, organization, and language use of the posted tasks on the class blog. The teacher monitored and checked the posted tasks, students’ comments, and gave general feedback to the whole class.

On the other hand, the same teacher covered the same topics and course content for the students of the control group. In fact, the same materials were used for these students except for the fact that they did not use educational blogging as the experimental group did. The three stages of process writing including drafting, redrafting, and editing the texts were applied for these students. Unlike the experimental group who had the opportunity to receive feedback outside the class *via* blogging, the students of the control group only received regular, face-to-face feedback inside the class and they could not use the Internet or technology devices inside the class. They did not receive peer feedback on their texts either and their feedback modality was traditional paper-and-pencil feedback. At the end of the course, the students of the two classes were given the posttests including the IELTS writing task and the L2 writing motivation scale.

### Results

To examine the contribution of the blog-based writing instruction on the writing skill of EFL learners and their writing motivation, the analysis of covariance (ANCOVA) was conducted to measure the effects of the two types of instructions employed in the control group and the experimental group on the dependent variables (i.e., writing performance and writing motivation).

Before inferential statistics (i.e., ANCOVA), descriptive statistics including means and standard deviations were computed. Then preliminary checks were made regarding the ANCOVA assumptions (e.g., normality, linearity, homogeneity of regression slopes; among others). The results indicated that the ANCOVA assumptions were not violated.

Regarding the effect of the blog-based writing instruction on the writing skill of EFL learners, as Table 1 shows, the mean score of the experimental group was raised from 56.11 (SD = 9.36) on the pre-test to 76.46 (SD = 8.72) on the post-test. Likewise, the mean score of the writing skill for the control group increased from 59.34 (SD = 9.42) on the pre-test to 68.91 (SD = 7.16) on the post-test. However, after adjusting for the pre-test scores, there was a statistically significant difference between the two groups on post-test scores of writing performance, [*F*(1,46) = 9.44, *p* = 0.004, partial eta squared = 0.170; see Table 2]. This result reveals that the EFL students of the experimental group enhanced their writing skill significantly more than the control group participants, suggesting that the blog-based instruction effectively enhanced the writing performance of the EFL students.

**Table 1:** Descriptive statistics.

	Group	*N*	Mean	Std. Deviation	Std. Error Mean
Pre-writing	Experimental	26	56.1154	9.36088	1.83582
	Control	23	59.3478	9.42245	1.96472
Post-writing	Experimental	26	76.4615	8.72803	1.71171
	Control	23	68.9130	7.16629	1.49428
Pre-motivation	Experimental	26	18.8077	4.59799	0.90174
	Control	23	19.0217	3.25604	0.67893
Post-motivation	Experimental	26	24.8654	4.08303	0.80075
	Control	23	21.1087	3.21911	0.67123

**Table 2:** ANCOVA results for writing skill.

Source	Type III sum of squares	df	Mean square	*F*	Sig.	Partial eta squared
Covariate (pre-test)	50.331	1	50.331	0.776	0.383	0.017
Between-subjects	612.452	1	612.452	9.441	0.004	0.170
Within-subjects	2983.957	46	64.869			

Concerning writing motivation, the descriptive statistics (see Table 1) shows that the experimental group’s mean score on writing motivation was 18.80 (SD = 4.59) in the pre-test and this value increased to 24.86 (SD = 4.08) in the post-test. Also, the writing motivation mean score for the control group was raised from 19.02 (SD = 3.25) on the pre-test to 21.10 (SD = 3.21) on the post-test. After adjusting for the pre-test scores of writing motivation, the results of ANCOVA (see Table 3) revealed that there was a substantial difference between the two groups in terms of writing motivation, [*F*(1,46) = 12.84, *p* = 0.001, partial eta squared = 0.231]. This outcome showed that blog-based writing instruction was useful in enhancing the writing motivation of the EFL participants.

**Table 3:** ANCOVA results for writing motivation.

Source	Type III sum of squares	df	Mean square	*F*	Sig.	Partial eta squared
Covariate (pre-test)	53.709	1	53.709	4.180	0.047	0.083
Between-subjects	177.353	1	177.353	13.803	0.001	0.231
Within-subjects	591.048	46	12.849			

## Discussion

The purpose of the current study was to investigate the usefulness of using blogs EFL writing instruction on writing performance and writing motivation of EFL students in a Chinese context. The findings indicated that blog-based writing instruction significantly improved L2 writing performance. The findings of this study are consistent with those of numerous other studies published in the literature (Montero-Fleta and Pérez-Sabater, 2010; Stevenson and Liu, 2010; Amir et al., 2011; Fageeh, 2011; Trajtemberg and Yiakoumetti, 2011; Wang and Vásquez, 2012; Vurdien, 2013; Chen, 2016; Hansen, 2016; Lee, 2020; Yousefifard and Fathi, 2021; Zhang and Zou, 2021; Gao et al., 2022). According to the findings of Arslan and Şahin-Kızıl (2010), the use of blog-based instruction helps students to get exposed to a huge number of various materials and writing input. Exposure to more language input possibly contributes to improving the writing performance of the EFL students. In other words, as a result of posting writing assignments on blogs, students can easily practice writing and be extensively exposed to writing input. The positive impacts of blogging on enhancing writing with extensive exposure to language input have been widely discussed by many researchers (Zhang, 2009; Wang and Vásquez, 2012; Lee and Markey, 2014; Chen, 2016). Moreover, according to Campbell (2003) blogging help learners have the opportunity to communicate with friends, give and receive feedback, and recognize their strengths and weaknesses in writing skill. The cooperative nature of blogs is in line with the concept of collaborative learning (Vygotsky, 1978) which states that learning is enhanced through peer work. With the help of blogging, students’ classroom limitations are removed as they can keep in touch with a huge number of audiences worldwide (Bransford et al., 2000; Bhattacharya and Chauhan, 2010; Sun, 2010). In addition to improving writing performance, blogging makes learners more self-reliant (Murray and Hourigan, 2008; Chen et al., 2011).

Furthermore, the outcomes revealed that participants’ writing motivation was significantly improved because of blogging. There is a strong relationship between the findings of this study and the reported findings in the literature (Montero-Fleta and Pérez-Sabater, 2010; Chen, 2016; Fathi et al., 2019; Rahimi and Fathi, 2021; Fan and Wang, 2022). As mentioned before, learning motivation is a combination of effort and desire to learn plus attitudes toward learning (Gardner et al., 1985), and writing motivation is essential for self-expression and communication among people (Chen, 2016). All these reasons possibly motivated the participants to make more prolonged efforts to enhance their L2 written performance. As mentioned by some researchers, students achieved satisfying outcomes and gained positive perceptions as a result of the increased motivation and confidence (Lin and Yang, 2011; Bikowski and Vithanage, 2016; Zhang and Zou, 2021). The use of blogging in the writing course might have encouraged the participants to enhance their self-regulatory strategies in L2 writing, acquire positive perceptions regarding L2 writing, boost their sense of competence in doing written tasks, and expect further success in L2 writing, all of which might have contributed to increasing writing motivation of EFL participants. According to the findings of a study by Ducate et al. (2011), technology enhanced students’ motivation to learn the target language as well as their confidence to write in a second language, enabling them to hold positive attitudes toward collaborative learning tasks. Furthermore, the same positive effects of technologies were observed by Bikowski and Vithanage (2016), who argued that improved motivation and confidence could be transferred to individual writing activities, thus resulting in improved writing skills.

## Conclusion and implications

Overall, the outcomes of this empirical study offered support for the effectiveness of blogs in improving the writing skills and writing motivation of Chinese EFL learners. A growing amount of L2 learning takes place outside of class, facilitated by technology (Lee, 2010). The findings of this study provide significant pedagogical implications as further support for the previously done bulk of research. For example, before implementing blog-based instruction, students’ knowledge of technology use should be evaluated, and accordingly, they must be provided with related training and instruction. Since many researchers reported that their participants had difficulty using technologies, it is not appropriate to assume all students are able to use them conveniently (Elola and Oskoz, 2010; Chao and Lo, 2011; Teo and Zhou, 2014; Zhang and Zou, 2021). As in a study done by Kessler (2009), students’ technological knowledge was assessed. In this study, students completed an online orientation, and their performance was analyzed by Kessler *via* screen-recording their mouse clicks. The researcher evaluated students’ abilities to use digital tools and access online resources. It is important for teachers to give students training on technology if they have difficulty using it before they implement specific instruction (Kessler, 2009; Li and Zhu, 2017; Li, 2018; Zhang and Zou, 2021). Trainers should provide learners with further instruction on blogging. Many authors have noted that technology enabled many “new” forms of collaborative writing not previously possible in traditional face-to-face settings, such as convenient drafting, planning, and revising (Cumming, 2009; Hsu and Lo, 2018; Zhang and Zou, 2021). Technologies may prove ineffective without related practices for using them and students may perceive them as less useful (Li and Zhu, 2017; Hsu and Lo, 2018; Zhang and Zou, 2021). Consequently, as a result of related training, students’ awareness and ability to write and collaborate could evolve along with technology as suggested by Kim and Cho (2017), Kessler (2009), and Zhang and Zou (2021). It is worth noting that applying blog-based instruction to guarantee the success of learning outcomes is not sufficient. The quality of writing and the level of interaction among students are significantly influenced by tasks that are linguistically and cognitively appropriate for L2 learners (Lee, 2008; Murray and Hourigan, 2008; Lee, 2010). Moreover, in order to encourage deeper reflection, instructors should guide students through peer commenting and provide them with strategies for critical thinking (Richardson, 2006; Murray and Hourigan, 2008; Lee, 2010).

## Limitations and suggestions for further research

Although the outcomes of this study provided a better understanding of how blogs can be used as a teaching tool for EFL instruction, further research is still needed to reach more conclusive results. With regard to the findings of this research, several limitations can be mentioned. First, since the participants had never already taken any blog-based writing courses, it may have been due to the Hawthorne effect that the participants were more motivated to participate in the writing class. Second, despite the fact that it was attempted to provide both groups with equal instruction, the experimental group had greater chance to revise their own composed tasks, view the essays of their peers, and share comments as well as feedback outside the classroom. This issue might make writing teaching for the two groups become unparalleled. This was so on the grounds that the asynchronous feature of the blogging approach allowed the experimental group students to expand their endeavors in having further writing practice beyond the classroom walls. Nevertheless, this limitation was partially mitigated by encouraging the control group students to carry out extra writing assignments outside the classroom. Additionally, the present study failed to account for computer anxiety’s intervening effects on the results of the study, despite the fact that all participants reported sufficient experience and familiarity with computers and technology devices before the study began. Also, investigating instructors’ opinions about blogs as a teaching tool, and using participants of different ages, genders, and levels of proficiency would be worthwhile. Finally, given the fact that the participants of this study were selected from the Chinese EFL context, the results may not be generalized to other EFL contexts. In light of the mentioned limitations, future researchers are invited to replicate such studies with larger number of participants from various age groups and selected from other EFL contexts. They also can investigate the role of technology anxiety and gender as the moderator variables in their studies. Last but not least, collecting qualitative data and uncovering students’ perceptions will help prospective researchers to shed more light on the findings and alleviate the potential problems caused by Hawthorne effect or unequal instructions.

## Data availability statement

The raw data supporting the conclusions of this article will be made available by the authors, without undue reservation.

## Ethics statement

The studies involving human participants were reviewed and approved by the Henan University of Animal Husbandry and Economy Research and Ethics Committee. The patients/participants provided their written informed consent to participate in this study.

## Author contributions

The author confirms being the sole contributor of this work and has approved it for publication.

## Funding

This study was supported by Humanistic and Social Science Youth Fund Project of Ministry of Education, “Research on Grammaticalization and Cognitive Motivation of Chinese and English Verbal Measurement Constructions” (grant no.: 20YJC740013), by Philosophical and Social Science Planning Project of Henan Province, “A Cognitive Contrastive Study of Chinese and English Non-prototypical Nominal Measurement Constructions”(grant no.: 2021BYY013), by Teacher Education Project of Henan Provincial Education Department, “Using Online Learning Resources to Promote English as a Foreign Language Teachers’ Professional Development in the Chinese Middle School Context” (grant no.: 2022-JSJYYB-027), and by Doctoral Research Start-up Fund Project of Henan University of Animal Husbandry and Economy (grant no. 2020HNUAHEDF047).

## Conflict of interest

The author declares that the research was conducted in the absence of any commercial or financial relationships that could be construed as a potential conflict of interest.

## Publisher’s note

All claims expressed in this article are solely those of the authors and do not necessarily represent those of their affiliated organizations, or those of the publisher, the editors and the reviewers. Any product that may be evaluated in this article, or claim that may be made by its manufacturer, is not guaranteed or endorsed by the publisher.
